# Supplementary material for: TRPM4 Blocking Antibody Protects Cerebral Vasculature in Delayed Stroke Reperfusion
Source: Biomedicines. 2023 May 19;11(5):1480. doi: 10.3390/biomedicines11051480 (PMC10216476; doi:10.3390/biomedicines11051480)
Supplement: Supplementary file 1 [file biomedicines-11-01480-s001.zip › biomedicines-2374797-supplementary.pdf]

Table S1. Summary of vascular areas (Figure 1B)

Summary of vascular areas			
**N (Images)**	**Hours after occlusion**	**Vascular areas (μm²)**	***p*-Value**
9	3	321.04 ± 43.98	<0.0001 3hr vs. 6hr, 9hr,12hr, 24hr
6	6	199.33 ± 25.07	< 0.001 6hr vs. 9hr,12hr, 24hr
6	9	89.54 ± 14.00	
8	12	87.86 ± 12.13	
8	24	86.80 ± 8.78	

Data are presented as mean ± S.E.M

Table S2. TRPM4 current density in control (Figure 2B) and ATP depletion (Figure 2D) condition

TRPM4 current density					
**IgG treatment**			**M4P treatment**		
**N**	**Current density (pA/pF)**	**Membrane potential (mV)**	N	**Current density (pA/pF)**	***p*-Value vs IgG**
15	-94.76 ± 21.44	-100 Control	12	-44.32 ± 10.17	0.041
15	155.47 ± 29.59	+100 Control	12	58.05 ± 8.51	0.004
12	-230.52 ± 61.01	-100 ATP depletion	9	-64.58 ± 28.10	0.039
12	266.28 ± 40.69	+100 ATP depletion	9	107.17 ± 29.62	0.008

Data are presented as mean ± S.E.M

Table S3. Summary of infarct area 24 hrs after operation (Figure 3D)

Summary of infarct area			
**N (Rat brains)**	**Treatment**	**Infarct area (%)**	***p*-Value**
8	M4P	11.80 ± 2.05	<0.01 M4P vs. Vehiclle, pMCAO
8	IgG	14.13 ± 1.52	
8	Vehicle	20.20 ± 1.27	
7	pMCAO	22.15 ± 1.97	

Data are presented as mean ± S.E.M

Table S4. Summary of the vascular diameter (Figure 4B)

Summary of the vascular diameter			
**N (Images)**	**Treatment**	**Vascular diameter (μm)**	***p*-Value**
14	pMCAO	5.02 ± 0.19	<0.0001 vs. IgG
14	IgG	6.13 ± 0.14	<0.05 vs. M4P
14	M4P	6.67 ± 0.10	

Data are presented as mean ± S.E.M

Table S5. Summary of Evens blue quantification (Figure 4D)

Summary of Evens blue quantification					
IgG treatment			M4P treatment		
N (Rat)	Evens blue (μg/g tissue)	Brain hemisphere	N (Rat)	Evens blue (μg/g tissue)	*p*-Value vs IgG
8	3.89 ± 0.66	Ipsi	10	2.40 ± 0.29	<0.05
8	1.25 ± 0.14	Contra	10	1.27 ± 0.14	

Data are presented as mean ± S.E.M

Table S6. Summary of cerebral blood flow (Figure 5C)

Summary of cerebral blood flow of 7 hr reperfusion					
IgG treatment			M4P treatment		
N (Rat)	Percentage of Baseline	Treatment	N (Rat)	Percentage of Baseline	*p*-Value vs IgG
6	24.22 ± 2.95	Occlusion	9	25.85 ± 2.13	
6	30.25 ± 5.25	Reperfusion	9	43.03 ± 3.83	<0.01

Data are presented as mean ± S.E.M

Table S7. Summary of cerebral blood flow (Figure 5D)

Summary of cerebral blood flow of 3 hr reperfusion					
IgG treatment			M4P treatment		
N (Rat)	Percentage of Baseline	Treatment	N (Rat)	Percentage of Baseline	*p*-Value vs IgG
10	31.22 ± 2.08	Occlusion	6	30.81 ± 2.74	
10	64.26 ± 6.98	Reperfusion	6	89.44 ± 8.22	<0.01

Data are presented as mean ± S.E.M

Table S8. Assessment of motor functions by Rotarod test (Figure 5E)

Rotarod test				
N (Rat)	Day after MCAO	Treatment	Percentage of Baseline	*p*-Value
7	1	Sham	86.27 ± 6.20	<0.001 vs. M4P, pMCAO, IgG for day 1
7	1	M4P	18.28 ± 7.35	
7	1	pMCAO	25.21 ± 4.28	
7	1	IgG	10.96 ± 4.70	
7	3	Sham	90.78 ± 4.87	<0.001 vs. M4P, pMCAO, IgG for day 3
7	3	M4P	52.86 ± 3.86	
7	3	pMCAO	46.02 ± 5.90	
7	3	IgG	30.67 ± 7.30	
7	5	Sham	93.28 ± 3.94	
7	5	M4P	84.09 ± 2.42	

7	5	pMCAO	59.29 ± 7.51
7	5	IgG	72.00 ± 7.32
7	7	Sham	95.89 ± 5.07
7	7	M4P	86.23 ± 3.65
7	7	pMCAO	75.01 ± 4.48
7	7	IgG	82.31 ± 3.09

Data are presented as mean ± S.E.M
